# Early Screening of Visual Processing Dysfunctions in Children Born Very or Extremely Preterm

**DOI:** 10.3389/fnhum.2021.729080

**Published:** 2021-11-01

**Authors:** Marlou J. G. Kooiker, Maud M. van Gils, Ymie J. van der Zee, Renate M. C. Swarte, Liesbeth S. Smit, Sjoukje Loudon, Sanny van der Steen, Irwin K. M. Reiss, Johan J. M. Pel, Johannes van der Steen

**Affiliations:** ^1^Department Neuroscience, Erasmus MC, Rotterdam, Netherlands; ^2^Royal Dutch Visio, Heerhugowaard, Netherlands; ^3^Department Child and Adolescent Psychiatry/Psychology, Erasmus MC-Sophia Children’s Hospital, Rotterdam, Netherlands; ^4^Royal Dutch Visio, Rotterdam, Netherlands; ^5^Division of Neonatology, Department Pediatrics, Erasmus MC-Sophia Children’s Hospital, Rotterdam, Netherlands; ^6^Division of Pediatric Neurology, Department Neurology, Erasmus MC-Sophia Children’s Hospital, Rotterdam, Netherlands; ^7^Department Pediatric Ophthalmology, Erasmus MC-Sophia Children’s Hospital, Rotterdam, Netherlands; ^8^Royal Dutch Visio, Huizen, Netherlands

**Keywords:** cerebral visual impairment (CVI), visual processing dysfunctions, preterm children, early screening, neurological risk, visual orienting functions, eye tracking

## Abstract

**Introduction:** Children with early brain damage or dysfunction are at risk of developing cerebral visual impairment (CVI), including visual processing dysfunctions (VPD), which currently remain largely undetected until school age. Our aim was to systematically screen for possible VPD in children born very or extremely preterm from 1 to 2 years corrected age (CA) and to evaluate the effectiveness of early referral.

**Method:** We included *N* = 48 children born < 30 weeks from 1 year CA. They underwent a two-step VPD screening based on (1) neurological signs indicative of visual brain damage evaluated by neonatologists and/or pediatric neurologist and (2) a functional assessment of visual orienting functions (VOF) with an eye tracking-based test. If at least one of these assessments was abnormal for their age, the children were classified as a risk of VPD and referred to undergo conventional visual diagnostics: ophthalmic exam and visual function assessment (VFA). At 2 years CA, VOF screening was repeated and neurodevelopment was assessed.

**Results:** 18 children (38%) were classified as at risk of VPD at 1 year CA. 7 children had abnormal neurological signs, 5 children had abnormal VOF, and 6 children had both. Subsequent ophthalmic exams (*N* = 14) showed severe hypermetropia in 21% and strabismus in 14%. VFA (*N* = 10) showed abnormal visual function and behavior in only 1 child. At 2 years CA, the total group showed an increase in abnormal VOF. Whereas the children at risk showed some normalization, the group without VPD risk at 1 year CA showed deterioration of VOF. Neurodevelopmental outcome did not clearly differ between risk groups.

**Conclusion:** Our findings show a substantial risk of VPD during visual screening (in 38%) at 1 year CA, but relatively few deficits on subsequent conventional ophthalmic exams and VFA. The data suggest that most conventional visual diagnostic methods at this young age are not related to the established VPD risks. VOF assessment should be used complimentary to these methods. The fact that at 2 years CA the number of children with a VPD risk based on abnormal VOF increased argues for more extensive and continuous screening in risk groups, at least until school age.

## Introduction

An increasing number of children has visual impairments due to brain damage or dysfunction, which is called cerebral visual impairment (CVI; Boot et al., 2010; Boonstra et al., 2012). There is currently no consensus on the exact definition of CVI: it is a broad and heterogeneous diagnosis that can include various types of visual dysfunction, depending on the underlying etiology (Ortibus et al., 2019). The problems children with CVI can experience already early in life range from lower-order visual sensory and oculomotor deficits to problems with information processing and higher-order visual perception problems (Dutton and Jacobson, 2001; Stiers et al., 2001). These problems can have a detrimental effect on (later) cognitive and motor development (Sonksen and Dale, 2002). An important aspect of CVI are visual processing dysfunctions (VPD): problems with detecting and processing incoming visual information, which has consequences for directing visual attention to specific locations, objects, or attributes within the visual scene. VPD are thought to reflect impairments in the intermediate stages of visual processing, mediated by both subcortical structures and primary and associative cortical visual areas (Martin et al., 1999). Most children at risk of CVI, and VPD, are enrolled in clinical follow-up programs from birth onward to monitor general health status, cognitive and motor neurodevelopment and for ophthalmic screening (e.g., retinopathy of prematurity, visual acuity deficits). However, no tests are included that monitor the functional impact that VPD may have on the first most critical years of development. The essence of the problem lies in the facts that (a) most tests for higher-level visual processing and/or perception require a certain degree of verbal communication between subject and assessor and (b), that mechanisms of brain maturation cause various higher-level visual functions to start and finish developing at differing ages, complicating their comprehensive assessment at a young age. As a consequence, the higher-level visual dysfunctions within the broad spectrum of CVI, i.e., VPD-related problems and perceptual dysfunction, generally start to be noticed when the functional consequences are beginning to affect social interactions or learning at school, i.e., around 5–6 years of age. It has been argued repeatedly that screening and possible intervention for CVI must take place preferably in the early years of high neuroplasticity, both by researchers (e.g., İdil et al., 2021) and clinicians (e.g., Federation Medical Specialists—CVI Guideline). Because of the methodological issues, many children at risk of CVI miss this window of opportunity. This may be overcome by early screening for VPD as an important hallmark of CVI in children.

When it comes to methods for VPD detection, there have been advances in the early detection of (a risk of) visual problems. Examples are assessment of basic visual functions in neonates as early as 31 weeks of gestation (Ricci et al., 2010), and a functional vision battery with cognitive and integrative aspects, to use between 1 and 4 years of age (Atkinson et al., 2002). These batteries involve various aspects of visual functions and rely on behavioral observations. In addition, a few computer-based methods have been developed, in which observation is combined with tracking the eyes of a child to assess visual orienting functions (VOF) as a proxy for visual attention (de Jong et al., 2016) and visual processing (Pel et al., 2010; Kooiker et al., 2016a). These methods rely on a close coupling between the visual attentional system and the oculomotor system (Munoz et al., 2007; Ross-Sheehy et al., 2017). As the nervous system develops, infant’s visual attentional capabilities rapidly develop during the first months in life, and this is apparent when observing simple orienting eye movements toward specific visual stimuli. Eye tracking-based testing can be done in a non-verbal manner to quantitatively assess various characteristics of VOF (compared to normative references), such as visual reaction times, fixation accuracies, and fixation durations. When combined with specific visual stimuli that are known to be separately processed in the brain’s visual system, VOF become a proxy for visual processing functions (Pel et al., 2010; Kooiker et al., 2016a).

In previous work, these VOF parameters proved reliable and valid not only in typically developing children, but also in heterogenous populations of children with (a high risk of) brain damage or dysfunction. VPD, as reflected by abnormal VOF, were particularly strongly correlated with signs of brain damage (i.e., visible damage on ultrasound or MRI scans) and a clinical diagnosis of CVI (Kooiker et al., 2014a) and were not related to visual acuity or oculomotor dysfunctions (Kooiker et al., 2014a; Pel et al., 2014). Moreover, abnormal VOF correlated with several aspects of visuoperceptual dysfunctions and daily visual problems in children with (suspected) CVI (Ben Itzhak et al., 2021). An important subgroup of children at risk are children born preterm, i.e., born before 37 weeks gestational age (GA). Because of improved neonatal health care, more preterm born children survive and grow up, even the children born very or extremely preterm, i.e., born before 30 weeks GA. The downside of this increased survival rate is the high risk of acquiring neurological damage with visual attention and processing abnormalities as a result (Atkinson and Braddick, 2007; Dutton, 2013). Previous studies using VOF parameters in children born very or extremely preterm showed abnormal VOF in the form of delayed viewing reaction times in 8–48%, that were related to structural brain damage around birth (van Gils et al., 2020), and to specific perinatal risk factors (Kooiker et al., 2019). Importantly, early VOF delays added to the prediction of later adverse neurodevelopmental outcome at 2 years corrected age (CA) in this population (Beunders et al., 2020).

Based on these recent insights, a logical next step is to use this eye tracking-based paradigm to screen children for risks of VPD 4–5 years earlier than is done in current practice. When this would be successful, it would bridge the gap between scientific findings and current visual (diagnostic) practice. To this end, we set up a prospective study to investigate the potential of early screening of VPD. Given the well-established notion that neuroplasticity is highest early in life, and the fact that other, low-level, visual screening methods showed to be feasible around 1 year of age, we chose to start screening for VPD at 1 year of age. This study is conducted in children born very or extremely preterm to constitute as a model for the larger group of children at risk of VPD due to prenatal or perinatal brain damage.

The aim of the present study was to explore whether systematic screening for possible VPD in children born very or extremely preterm from 1 year CA leads to an accurate selection and whether the age of 1 year is the most optimal age for screening. The following two research questions were addressed:

(1) Do abnormalities found in the early screening of VOF relate to the results of conventional visual diagnostics and daily visual problems at 1 year CA?
(2) How do early VPD risks develop over the course of 1 year and what is their implication for visual and neurodevelopment at 2 years CA?

## Materials and Methods

### Participants

All children who were born before 30 weeks GA and who participated in the outpatient clinical follow-up program of the dept. Neonatology, Erasmus MC-Sophia Children’s Hospital, were eligible for inclusion at 1 year CA. Children were excluded from participation based on the following criteria: Visual acuity below 0.05 Snellen equivalent, to ensure visibility of the eye tracking-based assessment; a high chance of epileptic activity during assessment, i.e., more than two attacks in the previous year or when actively using the anti-epileptic Vigabatrin (which may lead to visual dysfunctions; Maguire et al., 2010); retinopathy of prematurity (ROP) of grade 3 or higher assessed by a pediatric ophthalmologist, to exclude severe causes of visual dysfunctions other than VPD. Children were included for participation after receiving written informed consent from their parents or caregivers. The assessments were scheduled to coincide with an existing appointment at the Neonatology outpatient clinic to minimize burden for children and parents. The present study has been approved by Medical Ethical Testing Committee (METC) of Erasmus Medical Center, Rotterdam (MEC-2016-724) and adhered to the tenets of the Declaration of Helsinki (2013) involving research with human subjects.

#### Demographics

The following information was extracted from the medical records: gender, gestational age (GA, in weeks), birth weight (BW, in grams).

#### Structural Brain Damage

We obtained cerebral magnetic resonance imaging (MRI) scans that were made at 30 weeks GA or when the child was medically stable (range 29–35 weeks GA) as a part of standard medical care. The MRI scans were scored on brain growth and damage using a modified version of the standardized scoring system of Kidokoro et al. (2013). This included scoring of cerebral white matter (CWM), cortical gray matter (CGM), deep gray matter (DGM), and cerebellum. A modification was made on myelination and thickness of the corpus callosum because of the poor quality of some of the MRI scans. Since the brain measurement are dependent on GA, each scan was age-corrected (George et al., 2017). For a detailed description and visualization of the scoring method and analysis we refer to previous work (van Gils et al., 2020).

#### Daily Life Functioning

Upon inclusion, parents were asked to complete the Participation and Activity Inventory (PAI-CY 0-2) (Elsman et al., 2017). This questionnaire was developed to identify and monitor the developmental and participation needs of visually impaired children. It is the only available patient-reported visual outcome measure for young children and has satisfactory psychometric properties (Elsman et al., 2020). The instrument comprises items that are categorized in seven domains: attachment, stimulus processing, visual attention, orientation, play, mobility, and communication. Two additional domains, from the first PAI-CY version, were also included because they were relevant for the present study, i.e., sensory functioning and parental concerns. Each item was scored on a 4-point Likert scale with the response options: (0) not difficult, (1) slightly difficult, (2) very difficult, and (3) impossible. An average score per child per category was calculated.

### Visual Processing Dysfunctions Screening at 1 Year Corrected Age and Risk Assessment

Screening of VPD consisted of (1) neurological signs indicative of visual brain damage and/or VPD and (2) a functional assessment of visual orienting functions (VOF), evaluated with an eye tracking-based test and compared to age-matched normative references. If at least one type of abnormal VOF (in terms of viewing reaction times; see *VOF assessment*) and/or at least one neurological risk factor was found, the child was classified as having a risk of VPD and they were referred to undergo conventional visual diagnostics.

#### Neurological Risk Assessment

Neonatologists and/or a pediatric neurologist examined the child’s medical history for the presence of neurological risk factors for VPD in the context of prematurity (Schalij-Delfos et al., 2000; Ramenghi et al., 2010; Dutton, 2013; Sayeur et al., 2015), i.e.,

• Evidence for moderate to severe brain damage on neonatal MRI scans;
• Cerebral palsy: unilateral, bilateral, hemiplegia, diplegia;
• Infantile strabismus or nystagmus;
• Deviating head circumference (>1 SD in 12 months).

#### Visual Orienting Functions Assessment

VOF were measured with an eye tracking-based assessment focused on visual attention and processing functions. All tests were performed by trained researchers. The children were seated in front of the 24-inch eye tracker monitor at a distance of approximately 60 cm, either independently, on the lap of their parent or in a pram. No-one received verbal instructions, nor were they restricted in their movements. All assessments were conducted in a quiet room with ambient light conditions and total test duration was approximately 15 min. A 5-point Likert scale was used to monitor the level of attention, fatigue and restlessness/mobility, with option (1) representing “not at all” and (5) representing “all the time.”

**FIGURE 1:**
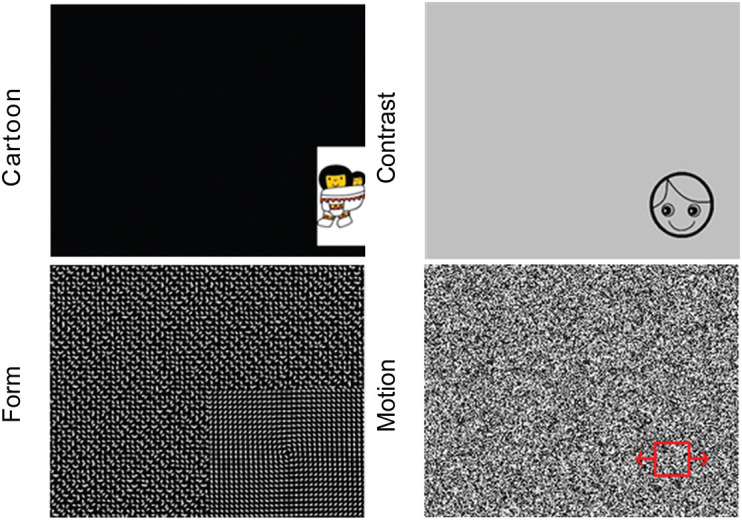
The four stimulus types with various visual content, from left to right and up to down: Cartoon, Contrast, Form, and Motion.

After a five-point calibration procedure, visual stimuli (images and movies) were presented on the monitor to engage reflexive orienting eye movements of the child, while simultaneously the eye positions were recorded using infrared cornea reflection (Tobii T60 XL or Tobii X3, Tobii Corporation, Danderyd, Sweden). That way, the child’s eye movement responses to various types of visual information with different salience levels (i.e., high-salient cartoons and contrast, moderate-salient motion and form; Figure 1) were automatically recorded. All stimuli were shown for 4 s. From the eye movement responses, various quantitative parameters were calculated to describe VOF per stimulus, i.e., the percentage of gaze data collected, the number of detected stimuli, the reaction time to fixation (RTF) of a stimulus and its individual variability RTvar. RTF is a measure for the timing of detecting, processing and executing an eye movement to the presented visual information and is the main study parameter (Pel et al., 2010; Kooiker et al., 2016a). In addition, for the high-salient cartoon stimulus two additional parameters were calculated: gaze fixation area (GFA), indicating the size of the fixated area in degrees and representing fixation accuracy; and the fixation duration (FD), indicating spontaneous duration of a child’s fixation on a stimulus. Individual parameter results were included when they adhered to the reliability criterion of > 25% of stimuli seen (Kooiker et al., 2014b). For children in whom calibration during the assessment failed, a post-calibration was performed prior to data analyses. For a detailed description of data processing and parameter analyses we refer to previous work (Kooiker et al., 2016a; van Gils et al., 2020).

The child’s VOF parameters per visual stimulus were compared with age-related normative data, i.e., developmental trajectories of an existing database of typically developing children born at term aged 0.5–1.5 years (baseline, *N* = 39) and aged 1.5–2.5 years (follow-up; *N* = 61). VOF parameter results were classified as normative or abnormal. For the parameters RTF and GFA this was based on the 95% confidence interval around the average (±2 SD) in the age-related norm group. For FD this was based on the 68% confidence interval (±1 SD) around the average of the norm group, because of the large variability in FD in the norm group.

### Referral of Children With a Visual Processing Dysfunctions Risk to Conventional Visual Diagnostics

The children who were identified as being at risk of VPD followed the conventional care pathway for children suspected of visual dysfunction. First, they underwent an orthoptic and ophthalmic exam at dept. Pediatric Ophthalmology, to evaluate visual acuity, refractive error and ocular alignment. This evaluation was performed by ophthalmologists and/or research orthoptists. Total duration of the exam was approximately an hour. Next, they were referred to a visual advisory and rehabilitation center to receive standard care, consisting of a visual function assessment (VFA) and, if applicable, a visual intervention program. With the VFA, the following visual sensory and oculomotor functions were assessed using a standardized protocol: ocular alignment and fixation preference, binocular vision, presence of nystagmus, oculomotor function (fixation, saccades, pursuit, motility), convergence, visual acuity, visual field, contrast sensitivity, and color vision. Performance per function was classified as normal or abnormal for the child’s age according to norm values per used test. For a detailed description of the assessment and classification per function (see Kooiker et al., 2016b). In addition, an observation of functional visual behavior was performed. The VFA assessments were performed by experienced orthoptists or optometrists and behavioral therapists. Together they determined the level of visual functioning of the child (Steendam, 2007), ranging from normal visual function to subnormal visual functioning (small degree of functional deficit, borderline), to profound visual dysfunction (clear deficits in visual attention and recognition), or legal blindness (only light responses). All assessments were performed according to a standardized protocol that ensured similar assessments, choice of tests and scoring by the various examiners.

### Follow-Up After 1 Year

One year after inclusion, i.e., at 2 years CA, in all included children (with and without VPD risk) the eye tracking-based VOF assessment was repeated. In addition, results from a neurodevelopmental assessment as part of the clinical follow-up of preterm born children were collected. The Bayley Scales of Infant and Toddler Development (BSID-III-NL) were performed by experienced (neuro)psychologists. The cognitive and motor scores and classifications were obtained as indications of overall neurocognitive and motor development, with higher scores indicating better development.

### Statistical Analysis

All data were tested for a normal distribution using the Kolmogorov-Smirnov test. This was significant for most parameters, therefore non-parametric tests were performed for the main analyses. Descriptive analyses were used to represent the presence and distribution of participant variables (i.e., demographics, structural data) and results of the VPD screening in the total study population.

To answer research question 1, Wilcoxon Signed-Rank tests were performed in the subgroup of children at risk of VPD, to compare results on the conventional visual diagnostic exams and the PAI-CY results. Additionally, these relations were explored for three different VPD risk groups (i.e., VOF risk, neurological risk, both risks). To answer research question 2, differences in VOF changes and neurodevelopmental outcome at 2 years CA were compared between the children with and without a VPD risk at 1 year CA, using Mann-Whitney *U*-tests. *P*-values < 0.05 were considered statistically significant.

## Results

### Participants

A total of *N* = 48 children born < 30 weeks GA were included at 1 year CA. Table 1 shows the demographics and clinical characteristics of the total group. Overall, there was a mild degree of structural brain damage around birth, indicated by a global score of 5 with the Kidokoro method.

**TABLE 1:** Participant characteristics.

**Characteristic**	***N* (%) or average (SD)**
Gender (boys)	30 (63%)
GA (weeks)	27.9 (1.5; range 24.6–29.9)
Birth weight (gr)	1,088 (246; range 565–1,550)
**Structural brain damage (range)**	
Global score (0–31)	4.54 (3.28)
CWM score (0–11)	2.64 (1.91)
CGM score (0–8)	0.23 (0.59)
DGM score (0–6)	1.57 (1.60)
Cerebellum score (0–6)	0.79 (0.80)

*SD, standard deviation; GA, gestational age; CWM, cerebral white matter; CGM, cerebral gray matter; DGM, deep gray matter.*

### Visual Processing Dysfunctions Screening at 1 Year Corrected Age

We found a high risk of VPD in 18 children (38%) at 1 year CA. In 7 children (15% of total) this was based on neurological risk factors, in 5 children (10% of total) this was based on abnormal VOF, and in 6 children (13% of total) both assessments were abnormal.

Table 2 shows the presence of neurological risk factors for VPD. Of the 13 children who had evidence of brain damage, in 8 children this was attributable to IVH. Other causes were germinal matrix hemorrhage (2 children), venous infarction (3 children), or ventricular dilation (2 children).

**TABLE 2:** The presence of neurological risk factors for VPD.

Type of neurological risk*	**Presence (*N*)**
Evidence of brain damage:	13
IVH grade II or III	8
Germinal matrix hemorrhage	2
Venous infarction	3
CNS sepsis	1
Ventricular dilation	2
Meningitis	1
Developmental venous anomaly	1
Cerebral palsy	4
Strabismus	3
Abnormal head circumference	0

*Factors are not mutually exclusive.

*IVH, intraventricular hemorrhage; CNS, central nervous system.*

Table 3 shows the overall results of the VOF assessment at 1 year CA. 94% of children had a successful calibration prior to testing. The attention, fatigue and mobility scores were all around 3; indicating scores slightly above average (“now and then”). The percentage of recorded data per stimulus ranged from 56% (Cartoon) to 69% (Motion). The percentage of stimuli seen ranged from 41% (Form) to 79% (Cartoon). The percentage of reaction times (RTs) that could be reliably calculated from all stimulus presentations classified as “seen” ranged from 54% (Cartoon) to 94% (Motion).

**TABLE 3:** General VOF results in the total group of preterm children at 1 year CA.

**Eye tracking feasibility factors**	***N* (%) or average (SD)**
Calibration successful	45 (94%)
Attention score (1–5)	3.46 (0.9)
Fatigue score (1–5)	2.98 (0.89)
Restless/mobility score (1–5)	3.10 (1.13)

**Overall viewing behavior per stimulus**	**Median [IQR]**

Cartoon % data	56 [32]
% seen	79 [30]
% RTs	54 [35]
Contrast % data	60 [29]
% seen	75 [25]
% RTs	75 [40]
Motion % data	69 [25]
% seen	75 [50]
% RTs	94 [32]
Form % data	60 [29]
% seen	41 [48]
% RTs	71 [73]

*SD, standard deviation; IQR, interquartile range; RT, reaction time.*

Table 4 shows the quantitative parameter results of the VOF assessment per visual stimulus for the total group at 1 year CA. In 12 children (25%) GFA was abnormal and in 7 children (15%) FD was abnormal. The percentage of abnormal RTF values ranged from 6% (3 children; Cartoons) to 13% (6 children; Contrast), and abnormal RTvar values ranged from 13% (6 children; Form) to 25% (12 children; Motion).

**TABLE 4:** Stimulus-specific VOF parameter values and the concurrent N and % of abnormal results compared to the age-matched normative references, in the total group of preterm children at 1 year CA.

**Stimulus parameter**	**Median [IQR]**	***N* (%) abnormal**
Cartoon GFA (deg)	1.98 [0.67]	12 (25%)
FD (ms)	1,304 [1,073]	7 (15%)
RTF (ms)	208 [76]	3 (6%)
RTvar (ms)	48 [39]	7 (15%)
Contrast RTF (ms)	432 [122]	6 (13%)
RTvar (ms)	42 [52]	7 (15%)
Motion RTF (ms)	707 [278]	5 (10%)
RTvar (ms)	97 [100]	12 (25%)
Form RTF (ms)	1,098 [450]	4 (8%)
RTvar (ms)	166 [152]	6 (13%)

*VOF, visual orienting functions; CA, corrected age; IQR, interquartile range; GFA, gaze fixation area; FD, fixation duration; RTF, reaction time to fixation; RTvar, reaction time variability.*

### Conventional Visual Diagnostics in Children With a Visual Processing Dysfunctions Risk

Table 5 shows the results of the ophthalmic exams and visual function assessments (VFA) that were performed after referral to conventional diagnostic services, separately for the type of VPD risk. Parents of 14 children (78% of the total VPD risk group) agreed with referral to the ophthalmologist. The ophthalmic exams showed moderate hypermetropia in 7 children (50%; considered normal at this age), severe hypermetropia in 3 children (21%), and strabismus in 2 children (14%). Rates of hypermetropia and strabismus were highest in the group with both VPD risk factors.

**TABLE 5:** Results of the ophthalmic exam and VFA after referral in the children at risk of VPD, separately for the different types of VPD risks (based on neurological factors, based on VOF, or based on both factors).

**Ophthalmic exam (*N* = 14)**	**Neurological risk (*N* = 4)**	**VOF risk (*N* = 4)**	**Both risk factors (*N* = 6)**
Moderate hypermetropia	3 (43%)	1 (20%)	3 (50%)
Severe hypermetropia	−	1 (20%)	2 (33%)
Strabismus	−	1 (20%)	1 (17%)

**VFA abnormalities (*N* = 10)**	**Neurological risk (*N* = 4)**	**VOF risk (*N* = 2)**	**Both risk factors (*N* = 4)**

No stereovision	1 (25%)	0	0
Nystagmus	0	0	0
Fixation	0	0	1 (25%)
Motility	0	0	0
Smooth pursuit	0	0	1 (25%)
Saccades	0	0	0
Convergence	0	0	0
Visual acuity mean (SD)	0.26 (0.11)	0.28 (0.32)	0.20 (0.09)
Visual acuity	0	0	0
Visual field	0	0	0
Contrast sensitivity	0	0	0
Color vision	0	0	0
Level of visual functioning			
Subnormal	−	−	1 (25%)
Normal	4 (100%)	2 (100%)	3 (75%)

*Factors are not mutually exclusive. VFA, visual function assessment; VPD, visual processing dysfunction; VOF, visual orienting functions; SD, standard deviation.*

Subsequently, parents of 10 children (56% of the total VPD risk group) agreed with referral to a visual rehabilitation center for an extensive VFA. Reasons for not agreeing with referral were that parents did not see any visual abnormalities in their child and/or that they already felt burdened with other medical appointments and assessments. In the group with a VPD risk based on neurological factors, one child did not have stereovision and average visual acuity was 0.26 decimal scale, which is normal for their age. Overall, the children in this group had normal levels of visual functioning for their age. In the group with a VPD risk based on abnormal viewing behavior, average visual acuity was 0.28 and both children had overall a normal level of visual functioning for their age. In the group with both VPD risk factors, average visual acuity was 0.20, slightly lower than in the other groups. One child showed abnormal fixation and smooth pursuit eye movements in combination with fluctuating viewing behavior (i.e., short fixations, many saccades) and as a result was classified with a subnormal level of visual functioning. The other children showed no abnormalities and had normal levels of visual functioning for their age.

### Daily Life Functioning

A total of 37 parents (77% of total group) completed the PAI-CY 0-2. Table 6 shows the results per category, separately for children not at risk and children at risk of VPD. Overall, the rate of daily life difficulties as indicated by parents in either group was relatively low. Nevertheless, compared to parents of children not at risk, parents of the children at risk of VPD gave higher scores for attachment, stimulus processing, orientation, mobility, communication, and parental concerns, indicating that more difficulties were experienced in these categories. Only the score for mobility was significantly higher (*U* = 86, *z* = −2.0, *p* = 0.043). The scores on visual attention, play, and sensory functioning where similar between the two groups.

**TABLE 6:** Results of the participation and activities inventory for children from 0 to 2 years (PAI-CY 0-2), separately for children not at risk and children at risk of VPD.

**PAI-CY category average score**	**Children not at risk of VPD, *N* = 24 (median[IQR])**	**Children at risk of VPD, *N* = 13, (median[IQR])**
Attachment	0 [0–0.5]	0.2 [0–0.4]
Stimulus processing	0 [0–0]	0 [0–0.4]
Visual attention	0.2 [0–0.4]	0.2 [0.1–0.4]
Orientation	0 [0–0]	0 [0–0.5]
Play	0.4 [0–1.0]	0.4 [0.2–0.4]
Mobility*	0 [0–0.6]	0.4 [0.3–0.8]
Communication	0 [0–0.5]	0.5 [0–1.0]
Sensory functioning	1.8 [1.6–2.2]	1.8 [1.5–2.0]
Parental concerns**	1.9 [1.6–2.1]	2.1 [1.8–2.3]

*Sign difference; **trend. VPD, visual processing dysfunctions.

### Follow-Up After 1 Year

A total of *N* = 43 children (90% of total group) repeated the VOF assessment at 2 years of age. With regard to changes in general VOF from 1 to 2 years CA, we found that scores on the eye tracking feasibility factors, i.e., calibration, attention, fatigue, and mobility, remained similar. In the total group, the percentage of data recorded decreased for Cartoon and remained similar for the other stimuli. The percentage of stimuli seen by children overall increased (ranging from + 0 to + 25%) and the percentage of calculated RTs remained the same. Compared to the children not at risk of VPD, in the children at risk of VPD the percentage of recorded data increased (ranging from + 4% to + 8%), the percentage of stimuli seen overall also increased (ranging from + 0 to + 38%), and the percentage of calculated RTs remained the same (Contrast and Motion) or increased (+2 to + 8%). However, none of these differences were statistically significant (Mann-Whitney *U*-tests all n.s.).

**FIGURE 2:**
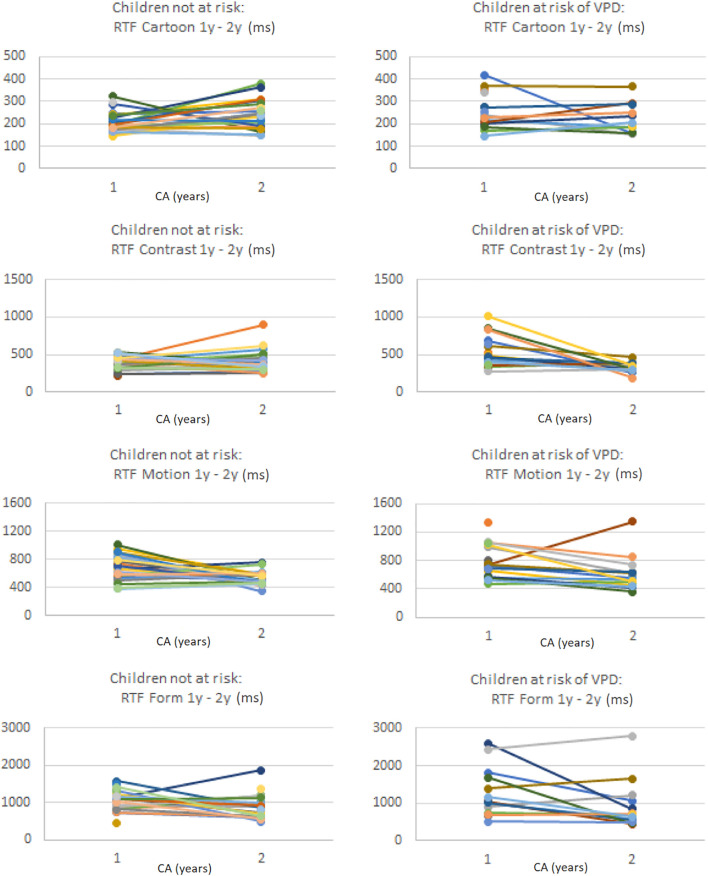
Per stimulus the RTF values (in ms) of individual preterm children at 1 year CA and at 2 years CA, separately for the children without a VPD risk **(left column)** and the children with a VPD risk **(right column)**. Note that values on the *y*-axis differ per stimulus.

Table 7 shows the median change in stimulus-specific VOF parameters and the change in the percentage of abnormal parameter values compared to age-based normative references, separately for the total group of included preterm children, the children not at risk of VPD at 1 year CA and the children at risk of VPD at 1 year CA. Figure 2 shows stimulus-specific RTF values of individual children, at 1 year CA and at 2 years CA.

**TABLE 7:** Median changes in stimulus-specific VOF parameters and changes in the percentage of abnormal parameter values compared to age-based normative references, separately for the total group, the children not at risk of VPD at 1 year CA and the children at risk of VPD at 1 year CA.

	**Total group (*N* = 43)**		**Children not at risk of VPD at 1 year CA**		**Children at risk of VPD at 1 year CA**	
	
**Stimulus**	**Median change**	**Change in% abnormal**	**Median change**	**Change in% abnormal**	**Median change**	**Change in% abnormal**
Cartoon GFA (deg)	+0.3	+10%	+0.22	+16%	+0.10	0%
FD (ms)	−138	+10%	+286	+7%	+538	+14%
RTF (ms)	+18	+9%	+53	+17%	+12	−6%
RTvar (ms)	−18	−8%	−17	−10%	−18	−5%
Contrast RTF (ms)	−63	−5%	−30	+13%	−129	−33%
RTvar (ms)	−6	−8%	−9	+6%	+3	−22%
Motion RTF (ms)	−184	+9%	−214	+10%	−162	+5%
RTvar (ms)	−22	−6%	−26	0%	+46	−11%
Form RTF (ms)	−169	+9%	−162	+17%	−425	−5%
RTvar (ms)	−15	−12%	+18	−17%	−104	−6%

*VOF, visual orienting functions; CA, corrected age; GFA, gaze fixation area; FD, fixation duration; RTF, reaction time to fixation; RTvar, reaction time variability.*

In the total group at 2 years CA, the percentage of abnormal GFA, FD, and RTF values relative to normative references increased compared to 1 year CA (except for RTF for Contrast), and RT variability decreased. Children who were not at risk of VPD at 1 year CA showed more changes to abnormal VOF parameter values at 2 years CA, whereas children with a VPD risk at 1 year CA showed more normalization (i.e., less abnormal parameter values compared to normative references) at 2 years CA. The decrease in absolute RTF value for Contrast was significantly larger in the group with a VPD risk compared to the group without a VPD risk (*U* = 93.5, *z* = −2.26, *p* = 0.024). The change in absolute RTvar value for Form was also significantly larger in the group at risk of VPD, where it considerably decreased (*U* = 19, *z* = −2.24, *p* = 0.025).

Specifically for RTF values we analyzed individual changes among all children with successful VOF measurements at both ages. For Cartoons, 3% had abnormal RTF at 1 year CA but no longer at 2 years CA, whereas 18% had normal RTF at 1 year CA but abnormal values at 2 years CA. For Contrast, Motion and Form, respectively 21, 2, and 13% had abnormal RTF values at 1 year but no longer at 2 years. Another 8, 20, and 23% had normal RTF values at 1 year but abnormal values at 2 years CA.

### Neurodevelopmental Outcome at 2 Years Corrected Age

Table 8 shows the results of the BSID-III-NL assessment, indicating the level of neurodevelopmental outcome, separately for the children not at risk of VPD and the children at risk of VPD. No differences were found between the groups on the cognitive and motor scores.

**TABLE 8:** Results of the Bayley Scales of Infant Development (BSID-III-NL) assessment (average, SD), separately for the children not at risk of VPD and the children at risk of VPD.

**BSID-III-NL category**	**Children not at risk of VPD**	**Children at risk of VPD**
Cognitive score	101 (10)	105 (7.8)
Cognitive classification	3.1 (0.6)	3.3 (0.5)
Motor score	99 (13)	99 (15)
Motor classification	3 (0.8)	3 (0.7)

*SD, standard deviation; VPD, visual processing dysfunction.*

## Discussion

The aim of the present study was to systematically screen for possible VPD at 1 year CA in children born extremely preterm (<30 weeks GA), to compare the outcome with conventional visual diagnostics and to evaluate the effectiveness of early referral at 2 years CA. We found a moderate risk of VPD during visual screening (in 38%) at 1 year CA on the account of abnormal neurological signs (15%), abnormal VOF (10%), or both risk factors (13%). Relatively few deficits were found on subsequent conventional ophthalmic examination and VFA. During follow-up at 2 years CA, the percentage of abnormal VOF results increased in the total group, indicating a larger number of children at risk of VPD at this age. Interestingly, this was not related to a VPD risk at 1 year CA.

### Screening at 1 Year Corrected Age

The innovative early screening at 1 year CA showed both neurological risks of VPD and functional risks based on abnormal VOF. The type of neurological VPD risk was predominantly based on IVH and other sorts of damage that are relatively common in children born extremely preterm (such as germinal matrix hemorrhages and venous infarctions). With regard to the functional, VOF-based risk assessment we supported with quantitative data that non-verbal eye tracking-based assessments are feasible in children born extremely preterm at this young age. The total group showed high degrees of successful calibration (94%) and average overall attention, fatigue and mobility/restlessness. Overall, we found very acceptable rates of general viewing behavior during the assessment: the percentage of recorded data ranged from 56 to 69%, the percentage of stimuli detected (“seen”) from 41 to 79%, and the percentage of RTs that could be reliably calculated out of seen stimuli from 54 to 94%. These parameters are all conditional factors for calculating the stimulus-specific quantitative VOF parameters. If a child has no attention for the test in general (% data), and subsequently does not detect the stimulus-specific target areas (% seen), then no RT can be calculated (% RTs).

The subsequent quantitative VOF analyses revealed that compared to age-matched norms, spontaneous fixation duration was abnormal in 15% of children and fixation accuracy was abnormal in 25%. The percentage of delays in VOF (abnormal RTF values) ranged from 6% (Cartoons) to 13% (Contrast), and high variability in VOF timing (abnormal RTvar values) ranged from 13% (Form) to 25% (Motion). These numbers are comparable to previous results in children born extremely preterm at 1 year CA, and minor differences can likely be attributed to sample size.

After referral of part of the risk group to conventional visual diagnostic services, relatively low levels of ophthalmic and VFA abnormalities were found at this age. Most abnormalities were found in the group with both VPD risk factors (i.e., neurological and VOF-based); where rates of hypermetropia and strabismus were highest, visual acuity was lowest and one child was classified with subnormal visual functioning based on abnormal oculomotor function and fluctuating viewing behavior. The VFA included a structured observation of the child’s visual and viewing behavior, which was expected to partly correlate with the eye tracking-based VOF assessment, as both revolve around active viewing and exploration. Surprisingly, in the children with abnormal VOF these abnormalities were not discovered by observation during VFA in most children. This may have to do with the fact that during observation, slighter deviations in viewing behavior are more difficult to notice than when using an automated recording system such as an eye tracker. As a consequence, most cases of abnormal visual function that were found with the screening were of an ocular origin. As expected beforehand, it is reflected by our data that most conventional visual diagnostic methods at this young age do not tap into cerebrally-mediated visual functions such as form- or motion processing. Whereas ophthalmic exams and VFA are necessary to map a child’s eye and visual function and deliver information on possible CVI signs such as visual field defects and crowding (see Federation Medical Specialists—CVI Guideline), other methods are needed to gather additional information on a VPD risk. Therefore, the available methods should be applied complementary to get a comprehensive overview of not only visual function but also of functional visual behavior.

Resulting from the relatively few abnormalities that were found with the conventional visual diagnostic exams, and given the relatively good levels of visual functioning in the group of children at risk of VPD, none of the children were in need of visual rehabilitation. In children born preterm at 5.5 years of age, it has also been shown that most did not qualify for visual rehabilitation services, despite some visual dysfunctions (Geldof et al., 2015). However, given the well-established risk of VPD in this population, the question remains whether visual function is indeed better than expected in this group or that these risks are just not sufficiently detected in clinical practice.

### Development of Visual Orienting Functions Over Time and Relation With Neurodevelopment

With regard to changes in general VOF from 1 to 2 years CA, we found that both the eye tracking reliability factors and overall viewing behavior remained largely similar. Interestingly, children with a risk of VPD at 1 year CA showed an increased percentage of recorded data and stimuli seen, compared to the children not at risk at 1 year CA. This indicates that overall attention for the test and detection of visual stimuli improved more in the VPD risk group.

With regard to the quantitative VOF parameters in the total group at 2 years CA, the percentage of abnormal GFA, FD, and RTF values relative to normative references increased compared to 1 year CA (except for RTF for Contrast), and RT variability decreased. This implies that the total group of preterm children at risk of VPD expanded at 2 years CA and confirms previous findings in a larger group of children born very and extremely preterm (Beunders et al., 2020; van Gils et al., 2020). Notably, the children with a VPD risk at 1 year CA showed more normalization of VOF parameters (i.e., decrease in the percentage of abnormal results), opposed to the group not at risk of VPD at 1 year CA who showed less function improvement, resulting in an increase in the percentage of abnormal results. However, the percentage of abnormal results fluctuated within and between the groups. This means that one screening at one particular age is not necessarily indicative. More specific, for the parameter RTF the percentage of children with normative values at 1 year CA but abnormal values at 2 years CA was highest for stimulus types that represent cerebrally-mediated visual processing, i.e., of motion and form information. For the parameter RTvar, indicating individual variability in viewing reaction times, we found a decrease in the percentage of abnormal results at 2 years CA. This is in accordance with normative developmental patterns where viewing behavior parameters become more stable and less variable with age. This also implicates that this specific parameter might be less sensitive to detect abnormalities in risk groups.

Taken together, these findings signal that performing a VOF-based VPD screening should be focused on the established parameters GFA, FD, and RTF. In addition, screening only at 1 year CA is not always indicative for later abnormal findings and is therefore not effective enough.

#### Neurodevelopmental Outcome

A disturbing question that is familiar to most caregivers of children at risk of VPD is which possible adverse events during development can be expected due to the neurological risks of their child. This warrants close monitoring of (neuro)developmental outcome in a range of domains. In our total study population, neurodevelopmental outcome in terms of overall cognitive and motor performance was quite good, in accordance with previous results (Beunders et al., 2020), and no differences were found based on an early VPD risk. The current neurodevelopmental results give a first indication at 2 years CA, but their meaning is restricted due to low sample size and the relatively low rates of objectified brain damage. However, in a recent larger study in 209 children of the same risk group, we showed that abnormal (delayed) VOF at 1 year of age significantly contributed to the prediction of adverse neurodevelopmental outcome at 2 years of age (Beunders et al., 2020). Other large cohort studies have shown that at 5 years of age, overall neurodevelopmental outcome of children born preterm is characterized by relatively high rates of severe and moderate disabilities, in multiple domains ranging from visual, auditory, and motor function to IQ, behavioral disorders and school assistance, irrespective of GA (e.g., Pierrat et al., 2021). Therefore, the signs of a relation of VOF abnormalities with abnormal neurodevelopmental outcome cannot be set aside and advocate for follow-up at later ages, i.e., at school age.

### Implications and Future Directions

One interpretation of the overall results is that the investigated screening for VPD at 1 year CA is not fully effective: not all risks were detected at this age, risk profiles considerably fluctuated after 1 year, and no clear relation with conventional visual diagnostic results or neurodevelopment was found. However, given that the risk group expanded at 2 years of age, it becomes apparent that screening may still be useful, albeit at different or various time points.

An important challenge was to determine the right age to start screening for VPD: when is early not too early? Because this is yet unknown, we chose to start at 1 year CA when basic visual and neurological development has completed and more elaborate and cerebrally-mediated developmental processes emerge (Ricci et al., 2010; Braddick and Atkinson, 2011). At present, we cannot answer the question which age is optimal for screening. However, from the fluctuating and variable VPD risks at 1 and 2 years of age in our study population, it follows that abnormalities can reveal themselves at multiple moments in development. In particular, VOF abnormalities in more basal or lower-order types of visual processing (to Cartoon and Contrast) seem to present earlier (i.e., higher rate of abnormalities at 1 year CA) than visual functions that are more cerebrally mediated, i.e., form and motion processing with increasing rates of abnormalities at 2 years CA. This is in accordance with knowledge on visual developmental trajectories (Braddick and Atkinson, 2011) and argues for a differential screening at 1 year and at 2 years. Possible developmental trajectories include normalization of early VPD risks until reaching school age after a couple of years, consistent delays compared to normative references, or an increase of VPD risk signs over time (i.e., “growing into deficit”). Moreover, our current results and previous work clearly show that, within the group of children born (extremely) preterm, there are certain subgroups with a considerably higher risk of VPD, namely the ones with other visual or ophthalmic conditions, evidence of or a high risk of brain damage (see also van Gils et al., 2020), certain perinatal risk factors related to hypoxia and/or pulmonary dysfunction (Boot et al., 2010; Kooiker et al., 2019). Particularly in these high risk groups it is advisable to accessibly follow further development through continuous yearly screening. This could be achieved by adding visual/VOF tests to existing clinical follow-up (programs) in other developmental domains, and by closely observing the type of risks and possible need for additional information on, e.g., brain imaging. That way, a more individual follow-up and profiling can be achieved. Promisingly, recent diagnostic developments at young ages have led to more advanced resources for structured observations (e.g., evidence-based history taking) and there are increasingly successful efforts of assessing early VPD performance on a task level, focused on visual perception instead of merely detection (Vancleef et al., 2020). This means that already from 3 years of age the possibilities for VPD/CVI tests are improving.

An important, yet open, question is how predictive early, relatively basal disruptions to the visual attention and processing system (mapped by VOF performance) are for later neurocognitive and higher-order visual dysfunctions (such as visual perception problems, impaired school performance in reading or writing). The VOF responses indicate whether and how fast children detect and fixate a specific stimulus, but are independent of (conscious) recognition. From recent work, we know that there are clear relations between VOF performance and visuoperceptual performance and daily life behavior in children with (suspected) CVI, mainly in the domains of visual (dis)interest, visual spatial perception and object processing (Ben Itzhak et al., 2021). This indicates that integrating VOF measurements into clinical screening procedures has added value as it incorporates aspects conditional to daily functioning and higher-order perception. Also, the presence of abnormal VOF may explain visual behavior such as a limited visual attention span and aberrant gaze behavior often seen in children at risk of CVI. This would be in accordance with guidelines such as the European perspective on CVI (Ortibus et al., 2019) and those published by the ophthalmic society in the Netherlands (Federatie Medisch Specialisten, 2019), that a CVI diagnosis is to be achieved through a multidisciplinary team, covering all previously mentioned aspects of the disorder. It may also add to circumventing the problems regarding the lack of a generally accepted definition on CVI, by focusing on a more operational definition concerning functional impairments in children that can be targets of (re)habilitation (Geldof et al., 2015).

From a research perspective, the next step is to comprehensively follow visual function and behavior up to school-age, both with functional VOF screenings and conventional neuropsychological assessments, to investigate the relation of VOF with the development of CVI-related symptoms and to determine the sensitivity and specificity of abnormal VOF findings for (future) CVI. If this early screening proves effective at school age, the age to start visual rehabilitation may be advanced and early visual development may be improved. Importantly, future research efforts should be directed toward developing VPD assessments to be used early in development.

In general, the ultimate goal is to achieve for all children at risk that when there are concerns about visual and viewing behavior, they enter the visual care chain as early as possible. Even though in many cases a definite diagnosis of CVI cannot be achieved before a certain age, the child and caregivers will benefit from early signaling clear signs of abnormalities that call for visual-developmental support.

## Study Limitations

Before the start of the study, it was difficult to estimate the number of children with a risk of VPD in this specific population of children born < 30 weeks at 1 year CA. This number turned out somewhat lower than expected and, along with recruitment and continuation issues, led to a low sample size, especially of the group with VPD risks. This prevented us from being able to statistically evaluate certain detailed questions, e.g., whether the heterogeneity of brain lesions influenced the results. Also, the study population is a relatively vulnerable risk group, particularly because of their young age. Recruiting at 1 year CA may still have been too early in the light of ongoing medical issues and difficulties for caregivers, explaining the relatively high drop-out rate. For all VOF parameters, it holds that the norm group at 1 year of age was smaller than that at 2 years CA, which may have influenced the percentage of abnormal results. It is therefore recommended to expand the youngest norm groups prior to clinical application of the method, preferably to month-based norms given the high rates of functional development at young ages.

Other drawbacks are that visual development was not followed at 2 years CA, as ophthalmic exams and VFA were not repeated. With this set-up we missed children in whom ophthalmic disorders started to present from 2 years of age. Lastly, the children with a risk of VPD based on abnormal VOF that started to emerge at 2 years CA (who did not have that risk at 1 year CA) were not referred to conventional diagnostics at all. All these limitations argue for a longer follow-up to determine the right age for VPD screening and VOF inclusion.

## Data Availability Statement

The raw data supporting the conclusions of this article will be made available by the authors, without undue reservation.

## Ethics Statement

The studies involving human participants were reviewed and approved by the Medical Ethics Testing Committee, Erasmus Medical Center, Rotterdam, NL. Written informed consent to participate in this study was provided by the participants’ legal guardian/next of kin.

## Author Contributions

JS, SS, and IR conceived the study. MK and JP designed the study and procedures. MK, MG, YZ, and JP interpreted the data. MK and MG drafted, revised, and prepared the manuscript. All authors gave final approval for the submitted manuscript and significantly contributed to procedures and execution.

## Conflict of Interest

The authors declare that the research was conducted in the absence of any commercial or financial relationships that could be construed as a potential conflict of interest.

## Publisher’s Note

All claims expressed in this article are solely those of the authors and do not necessarily represent those of their affiliated organizations, or those of the publisher, the editors and the reviewers. Any product that may be evaluated in this article, or claim that may be made by its manufacturer, is not guaranteed or endorsed by the publisher.

## Abbreviations

CA: corrected age
CVI: cerebral visual impairment
GA: gestational age
FD: fixation duration
GFA: gaze fixation area
METC: medical ethical testing committee
MRI: magnetic resonance imaging
PAI-CY: participation and activities inventory - children and youth
ROP: retinopathy of prematurity
RT: reaction time
RTF: reaction time to fixation
VFA: visual function assessment
VOF: visual orienting functions
VPD: visual processing dysfunctions.
